# Urinary Concentrations of Triclosan, Benzophenone-3, and Bisphenol A in Taiwanese Children and Adolescents

**DOI:** 10.3390/ijerph14121545

**Published:** 2017-12-10

**Authors:** Fu-Kuei Chang, Jentaie Shiea, Hsin-Jen Tsai

**Affiliations:** 1 Department of Health Management, College of Medicine, I-Shou University, Kaohsiung 82445, Taiwan; hjtsai@isu.edu.tw; 2 Department of Chemistry, College of Science, National Sun Yat-Sen University, Kaohsiung 80424, Taiwan; jetea@mail.nsysu.edu.tw

**Keywords:** triclosan, benzophenone-3, bisphenol A, children, adolescents

## Abstract

The purpose of this study was to determine the levels of urinary triclosan (TCS), benzophenone-3 (BP-3), and bisphenol A (BPA) in 52 children and 71 adolescents. The effects of age and sex on the levels of urinary TCS, BP-3, and BPA were explored, respectively. Results demonstrated the overall detection rates of urinary TCS, BP-3, and BPA were 18.7%, 8.1%, and 49.6%, respectively. The females had higher TCS concentrations than males (*p* = 0.051). The detection rate of urinary BP-3 in females (12.3%) was higher than that in males (0%) (*p* = 0.015). Moreover, the detection rate of urinary BP-3 in adolescents (14.1%) was higher than that in children (0%) (*p* = 0.005). For children, no urinary BP-3 was found. There were no differences in detection rates and concentrations of urinary TCS, BP-3, and BPA between males and females, respectively. For adolescents, urinary BP-3 was only found in the females. Urinary TCS levels in females were higher than those in males (*p* = 0.047). The present study showed that urinary TCS concentrations in females were significantly higher than those in males, respectively. In addition, BP-3 was only detected in urine samples of female adolescents. Sex and age were the important factors influencing urinary TCS and BP-3 concentrations.

## 1. Introduction

Humans are potentially exposed to several phenolic chemicals in commonly used products that may cause endocrine-disrupting effects. For example, triclosan (2,4,4′-trichloro-2′-hydroxy-diphenyl ether, (TCS) has been added to soaps, toothpastes, mouthwashes, deodorants, and cosmetics as a preservative and antiseptic agent [1,2]. Bisphenol A (2,2-bis(4-hydroxyphenyl)propane, (BPA) has been used in the production of polycarbonate plastics and epoxy resins. It can be found in water bottles, baby bottles, plastic dinnerware, dental sealants, and the linings of canned food containers [3,4,5]. Additionally, some thermal paper and polyvinyl chloride plastics can also contain low levels of bisphenol A [6,7]. Benzophenone-3 (2-hydroxy-4methoxybenzophenone, (BP-3) is a common ingredient in sun-blocking agents and is commercially synthesized as a sunscreen for use in lotions, conditioners, and cosmetics [8,9].

Oral and dermal absorption are the major routes of exposure of these phenolic chemicals. Due to their extensive use, the general population is potentially at risk of exposure to TCS, BPA, and BP-3, which result in their wide distribution in human blood, breast milk, and urine, respectively [10,11,12,13,14,15,16,17,18,19,20]. TCS, BPA, and BP-3 have also been detected in a variety of environmental substances, including water, wastewater, river, and sediment [21,22,23,24,25,26,27,28,29].

TCS, BPA, and BP-3 have been reported for endocrine-disruption potentials in a number of in vitro and in vivo studies. In general, BPA and TCS are considered to have a weak estrogenic effect [30,31], whereas BP-3 was determined to be slightly estrogenic or to have anti-androgenic properties [32,33,34].

Several studies by Howdeshell et al. (1999) indicated that BPA altered the postnatal growth rate and reproductive function in female mice [35,36,37,38]. Low doses of BPA possibly altered the development of the fetal prostate and mammary gland, and decreased efficiency of sperm production in mice [39,40,41,42]. TCS was likewise found to inhibit thyroid hormone-associated gene expression and postembryonic anuran development in tadpoles [43]. Kumar et al. [44] reported that the levels of serum luteinizing hormone, follicle stimulating hormone, pregnenolone, and testosterone were significantly diminished in TCS treated rats.

Coronado et al. [45] have suggested that reproduction and hatching success were reduced in BP-3 treated Japanese medaka, respectively (Oryzias latipes). Vitellogenin induction (Oncorhynchus mykiss) was observed in BP-3 treated male Japanese medaka and rainbow trout, respectively. Low levels of BP-3 also inhibited steroidogenesis and affected hormonal pathways in male zebrafish [46].

These phenols are also suspected to affect development in humans. Philippat et al. [47] indicated that urinary concentrations of BP-3 were positively associated with weight and head circumference at birth. Urinary BPA concentrations were positively associated with head circumference. Wolff et al. [48] reported that higher maternal BP-3 concentrations were associated with a similar decrease in birth weight among girls but with greater birth weight in boys. Koeppe et al. [49] observed a positive association between urinary TCS concentrations and serum total triiodothyonine (T3) levels in adolescents. Tsutsumi [50] reported that serum BPA concentrations were significantly higher in normal men and in women with polycystic ovary syndrome than in normal women, respectively. No hormonal changes were observed in 32 healthy volunteers during four days of application of 10% BP-3 lotion [51].

However, there are likely to be some important variables (e.g., age and sex) that determine whether people contact TCS, BP-3, and BPA-containing products. Relative children and males, adolescents, and females have more chances to use personal care products such as toothpastes, lotions, and cosmetics. The purpose of this study is to explore the effects of age and sex on the levels of urinary TCS, BP-3, and BPA. The elementary school children and college students were selected as our study subjects.

## 2. Materials and Methods

### 2.1. Chemicals and Reagents

Triclosan (TCS), bisphenol A (BPA), and benzophenone-3 (BP-3) standards were all purchased from Sigma-Aldrich (St. Louis, MO, USA). HPLC-grade methanol and water, analytical-grade ammonium acetate, formic acid, andβ-Glucuronidase/arylsulfatase were provided by Merck (Darmstadt, Germany). ^13^C_12_-triclosan was obtained from Cambridge Isotope Laboratories Inc. (Andover, MA, USA).

### 2.2. Sample Collection

Morning urine samples were obtained from 52 elementary school children of 11–13 years old and 71 college students of 19–21 years old in southern Taiwan, respectively. Written informed consent was obtained from each subject and their parents. The study procedures were approved by the Institutional Review Board of I-Shou University (ISU-IRB-98-03).

### 2.3. Sample Preparation

The pretreated and analyzed methods of urine samples were referred to in the procedures, as reported by Ye et al. [52]. At first, urine samples were thawed at room temperature and vortex mixed. Next, a 100 µL aliquot of urine was transferred into a tube and spiked with 50 µL of ^13^C_12_-Triclosan. After gentle mixing, samples were added to 50 µL of glucuronidase/sulfatase solution (in a 0.5-M ammonium acetate buffer, pH 5.0), and the hydrolysis was allowed to proceed at 37 °C overnight. After the incubation, the deconjugated urine sample was diluted with 800 µL to 0.1 M formic acid and centrifuged. The aliquot was injected into the on-line solid phase extraction (SPE) coupled to high-performance liquid chromatography (HPLC)–tandem mass spectrometry (MS/MS).

### 2.4. On-Line SPE-HPLC-MS/MS

The on-line SPE-HPLC-MS/MS system was constructed from several Agilent 1290 modules coupled to a triple quadrupole Agilent 6430 mass spectrometry (Agilent Technologies, Santa Clara, CA, USA). The mass spectrometer was equipped with an electrospray ionization (ESI) interface. The SPE column was a LiChrosphere RP-18 ADS (25 × 4 mm, 25 µm particle size, 60-Å pore size, Merck KGaA), and the HPLC column was ZORBAX Eclipse XDB-C18 (150 × 4.6 mm; Agilent). Methanol and water were used as mobile phases. The procedure for extracting the analytes from the urine involved concurrent SPE and HPLC-MS/MS cycles (Table 1).

The Agilent 6430 mass spectrometry was used in negative mode with the following settings: source gas (nitrogen) with a flow rate of 10 L/min at a temperature of 350 °C, nebulizer pressure of 50 psi, and capillary voltage of 4 kV. The mass spectrometer operating parameters were optimized using Agilent Optimizer Software for the analytes (Table 2). The limit of detection (LOD) of the analyte was calculated based on the standard deviation (SD) of seven replicates of the spiked sample. The LOD was defined as 3 times the SD. Recoveries for TCS, BPA, and BP-3 were 104 ± 5% (mean ± SD), 104 ± 6%, and 101 ± 5%, respectively. The LOD of TCS in the present study was 1.56 μg/L, which was similar to those reported at 2.27–2.3 μg/L in some studies [10,12], but substantially higher than those used in other studies at 0.9 ng/L–0.06 μg/L [53,54,55]. The LOD of BP-3 in the present study was 0.16 μg/L, which was similar to those reported at 0.07–0.34 μg/L in some studies [12,18,54]. The LOD of BPA in the present study was 0.16 μg/L, which was similar to those reported at 0.05–0.4 μg/L in some studies [12,13,14,54,55,56,57], but substantially lower than that used in a study at 0.5 ng/L [53].

### 2.5. Statistical Analysis

The data were analyzed using the SPSS 18.0 statistical package. To demonstrate the distribution and disparity of urinary TCS, BPA, and BP-3 levels, only positive samples > LOD were used to calculate the geometric means and ranges, respectively. Mann-Whitney U test was used to evaluate the differences of urinary TCS, BPA, and BP-3 levels between males and females, and between children and adolescents, respectively. Chi-square/Fisher’s exact test was used to compare the differences of detection rates of urinary TCS, BPA, and BP-3 between males and females, and between children and adolescents, respectively. The *p*-value of <0.05 was considered statistically significant for these tests.

## 3. Results

The overall detection rates of TCS, BP-3, and BPA were 18.7%, 8.1%, and 49.6%, respectively. Concentrations of TCS, BP-3, and BPA ranged from <1.56 to 198.66 µg/L, <0.16 to 97.02 µg/L, and <0.16 to 190.10 µg/L, respectively. The geometric mean (GM) concentrations of urinary TCS, BP-3, and BPA in the detected samples were 59.52 µg/L (n = 23), 19.72 µg/L (n = 10), and 5.81 µg/L (n = 61), respectively. The present study observed that the females had higher TCS concentrations than the males (*p* = 0.051). The detection rate of urinary BP-3 in females (12.3%) is higher than that in males (0%) (*p* = 0.015). In addition, the detection rate of urinary BP-3 in the college students (14.1%) is also higher than that in elementary school students (0%) (*p* = 0.005) (Table 3).

For elementary school children, the detection rates of TCS and BPA were 13.5% and 48.1%, respectively. No urinary BP-3 was found. Concentrations of TCS and BPA ranged from <1.56 to 149.64 µg/L, and <0.16 to 22.27 µg/L, respectively. The GM concentrations of urinary TCS and BPA in the detected samples were 61.09 µg/L and 5.61 µg/L, respectively. No differences of levels and detection rates of urinary TCS, BP-3, and BPA between males and females were found, respectively.

For college students, the detection rates of TCS, BP-3, and BPA were 22.5%, 14.1%, and 50.7%, respectively. Urinary BP-3 was only found in the females. Concentrations of TCS, BP-3, and BPA ranged from <1.56 to 198.66 µg/L, <0.16 to 97.02 µg/L, and <0.16 to 190.10 µg/L, respectively. The GM concentrations of urinary TCS, BP-3, and BPA in the detected samples were 58.84 µg/L, 19.72 µg/L, and 5.95 µg/L, respectively. Urinary TCS levels in the females were higher than those in the males for college students (*p* = 0.047).

## 4. Discussion

In the present study, the detection rates for TCS (18.7%) and BP-3 (8.1%) were lower than those available in the literature (74.6–100% and 85.3–98.4%) [10,12,53,54,55]. The detection rate of BPA in the present study was 49.6%, which was similar to that reported at 50% in a study [57], but substantially lower than those used in other studies at 77.5–100% [12,13,14,54,55,56]. In this work, we studied human exposure to TCS, BP-3, and BPA in Taiwan, a country where, to our knowledge, there had been consumer education program caution against TCS, BP-3, and BPA and restrictions on their use over the last decade by Consumers’ foundation. Consumers’ foundation reported that TCS, BP-3, and BPA were suspected of affecting human hormones. People who were concerned about TCS, BP-3, and BPA exposures from personal care products could have avoided using products that contained or released TCS, BP-3, and BPA. This was one of the reasons why the detection rate for TCS, BP-3, and BPA in the present study is almost lower than those available in the literature. Our previous study [23] also showed that TCS concentrations in surface water samples of Rivers in Taiwan (<LOQ (limit of quantitation)–31.1 ng/L) were lower than the ranges reported for rivers in China (Zhujiang River 90.2–478 ng/L) [58] and US streams (<LOQ–2.3 μg/L) [21]. In addition, the other reasons for lower detection rates included LOD, sample size, and different cultural and living habits.

For urinary TCS of the detected samples, the present study observed that the females in the college had significantly higher TCS concentrations than the males (*p* = 0.047). This observation was consistent with the findings of Li et al. [53] that the concentrations of urinary TCS in females were higher than those in males. The females might pay more attention to their appearances and have better hygiene habits. For example, daily applying cosmetics and brushing teeth twice a day were likely to increase chances to contact TCS-containing products.

The medians of TCS of 52 children of 11–13 years old (<1.56 μg/L) and 71 adolescents of 19–21 years old (<1.56 μg/L) in the present study were lower than those for 314 children of 6–11 years old (5.9 μg/L) and 715 adolescents of 12–19 years old (10.2 μg/L) in the study of Calafat et al. [10], respectively. Other studies showed the geometric mean (GM) of TCS for 90 girls aged 6–9 years old (10.9 μg/L) [12], 20 Belgian adolescents (1.71μg/L) [59], 95 primary school students aged 7–12 years old (7.52 μg/L), 64 college students aged 18–24 years old (3.03 μg/L) [53], and 129 children and adolescents aged 6–21 years old (1.45μg/L) [54], respectively.

For urinary BP-3 of the detected samples, the present study observed that the females had a significantly higher detection rate of urinary BP-3 than the males (*p* = 0.015). No urinary BP-3 was found in the males. This was consistent with the findings of Calafat et al. [18] that the concentrations of urinary BP-3 in females were higher than those in males. The females might have had higher frequencies to applicate BP-3 lotion to avoid sun exposure. In addition, the present study also observed that no urinary BP-3 was detected in elementary school children. This showed that the frequencies of sunscreen application in adolescents were higher than those in children.

The medians of BP-3 of 52 children of 11–13 years old (<0.156 μg/L) and 71 adolescents of 19–21 years old (<0.156 μg/L) in the present study were lower than those for 314 children aged 6–11 years old (17.2 μg/L) and 715 adolecents aged 12–19 years old (20.0 μg/L) in the study of Calafat et al. [18], respectively. Other studies showed the GM concentration of BP-3 for 90 girls aged 6–9 years old (19.7 μg/L) [12] and 129 children and adolescents aged 6–21 years old (1.41 μg/L) [54], respectively.

The sex-related differences of urinary BPA from the findings of previous studies were not all consistent. He et al. [57] reported that urinary BPA concentrations in females were significantly lower than those in males in 952 Chinese populations. On the contrary, urinary BPA levels were found to be significantly higher in females than in males for 2517 U.S. populations [14]. In the present study, there was no significant difference in urinary BPA levels between females and males, which was consistent with the finding in a particular study [60]. Kim et al. [60] reported urinary BPA levels were similar in 15 men and 15 women that were aged about 43 years old.

The medians of BPA of 52 children of 11–13 years old (<0.156 μg/L) and 71 adolescents of 19–21 years old (<0.156 μg/L) in the present study were similar to those for 922 Chinese population (<LOD μg/L) in the study of He et al. [57], but lower than those for 314 children aged 6–11 years old (3.7 μg/L) and 715 adolescents aged 12–19 years old (4.2 μg/L) in the study of Calafat et al. [14], 394 U.S. population (adults) (1.28 μg/L) in the study of Calafat et al. [13], and 1870 Korean population aged 18–69 years old (2.07 μg/L) in the study of Kim et al. [55], respectively. Other studies showed the GM concentration of BPA for 90 girls aged 6–9 years old (2.0 μg/L) [12], 20 Belgian adolescents (1.25 μg/L) [59], 95 primary school students aged 7–12 years old (4.10 μg/L), and 64 college students aged 18–24 years old (3.03 μg/L) [53], and 129 children and adolescents aged 6–21 years old (1.37μg/L) [54], respectively.

## 5. Conclusions

The present study showed that urinary TCS and BP-3 concentrations in females were significantly higher than those in males, respectively. In addition, urinary BP-3 was only detected in the female samples of college students. Sex and age were the important factors influencing urinary TCS and BP-3 concentrations, respectively. Further studies are needed to assess the effects of these phenol chemicals on human health, especially for more highly exposed groups.

## Figures and Tables

**Table 1:** Concurrent solid phase extraction (SPE) clean up and high–performance liquid chromatography–tandem mass (HPLC/MS/MS) analysis time line.

Period				1	2	3		4		5	6
Time (min)			0	0.1–2	2–7	7.01–11		11–17		17–18.5	18.5–23.5
**SPE of Sample N + 1**			Start	Analyte Transfer and dilution	Regenerate SPE column	Equilibrate SPE column		Sample loading		SPE Column Wash	Stop Pump1
	Autosampler Valve		1–2	1–2	6–1	6–1		1–2		6–1	1–2
	Pump1	mL/min	0	0.25	1.5	1.0		1.0		1.0	0
		MeOH%		20	100	20		20		20	20
**HPLC of Sample N**				Analyte Transfer	HPLC separation and MS/MS acquisition						Equilibrate Pump2
	Ten-port valve		10–1	1–2	10–1						10–1
	Pump2	mL/min	0.5	0.5	0.75 HPLC gradient elution						0.5
					Time (min)	2	8		17	18.5	
		MeOH%	50	50							
					MeOH%	50	100		100	100	

**Table 2:** MS/MS transitions and parameter for analytes and internal standard.

Analytes	Precursorion (*m*/*z*)	Production (*m*/*z*)	Fragmentor (V)	Collision Energy (eV)
Triclosan	288	142	20	32
Bisphenol A	227	212	100	24
Benzophenone-3	227	211	120	20
^13^C_12_-Triclosan	299	148	35	36

**Table 3:** Detection rates (%) and concentrations (µg/L) of urinary TCS, BP-3, and BPA in primary school children and college students by sex and age subgroups.

	Elementary School Children (n_male_ = 30, n_female_ = 22)		College Students (n_male_ = 12, n_female_ = 59)		Total (n_male_ = 42, n_female_ = 81)	
Male	Detection rate	GM (range)	Detection rate	GM (range)	Detection rate	GM (range)
TCS	13.3	55.49 (28.38–127.28) *	41.7	42.98 (36.98–54.18)	21.4	48.15 (28.38–127.28)
BP-3	0	-	0	-	0	-
BPA	50.0	5.84 (2.86–12.72)	41.7	6.24 (2.86–15.18)	47.6%	5.94 (2.86–15.18)
Female						
TCS	13.6	69.43 (47.30–149.64)	18.6	67.87 (30.96–198.66)	17.3	68.21 (30.96–198.66)
BP-3	0	-	16.9	19.72 (7.14–97.02)	12.3	19.72 (7.14–97.02)
BPA	45.5	5.29 (2.55–22.27)	52.5	5.91 (1.94–190.10)	50.6	5.75 (1.94–190.10)
Overall						
TCS	13.5	61.09 (28.38–149.64)	22.5	58.84 (30.96–198.66)	18.7	59.52 (28.38–198.66)
BP-3	0	-	14.1	19.72 (7.14–97.02)	8.1	19.72 (7.14–97.02)
BPA	48.1	5.61 (2.55–22.27)	50.7	5.95 (1.94–190.10)	49.6	5.81 (1.94–190.10)

* only positive samples > limit of detection (LOD) were used to calculate the geometric mean and range.

## References

[1] Bhargava H.N., Leonard P.A. (1996). Triclosan: Applications and safety. Am. J. Infect. Control.

[2] Jones R.D., Jampani H.B., Newman J.L., Lee A.S. (2000). Triclosan: A review of effectiveness and safety in health care settings. Am. J. Infect. Control.

[3] Arenholt-Bindslev D., Breinholt V., Preiss A., Schmalz G. (1999). Time-related bisphenol-A content and estrogenic activity in saliva samples collected in relation to placement of fissure sealants. Clin. Oral Investig..

[4] Howe S.R., Borodinsky L., Lyon R.S. (1998). Potential exposure to bisphenol a from food-contact use of epoxy coated cans. J. Coat. Technol..

[5] Sajiki J., Yonekubo J. (2003). Leaching of bisphenol A (BPA) to seawater from polycarbonate plastic and its degradation by reactive oxygen species. Chemosphere.

[6] Biedermann S., Tschudin P., Grob K. (2010). Transfer of bisphenol A from thermal printer paper to the skin. Anal. Bioanal. Chem..

[7] Yamamoto T., Yasuhara A. (1999). Quantities of bisphenol A leached from plastic waste samples. Chemosphere.

[8] Gonzalez H., Farbrot A., Larkö O., Wennberg A.M. (2006). Percutaneous absorption of the sunscreen benzophenone-3 after repeated whole-body applications, with and without ultraviolet irradiation. Br. J. Dermatol..

[9] Rastogi S.C. (2002). UV filters in sunscreen products—A survey. Contact Derm..

[10] Calafat A.M., Ye X., Wong L.Y., Reidy J.A., Needham L.L. (2008). Urinary concentrations of triclosan in the U.S. population: 2003–2004. Environ. Health Perspect..

[11] Allymr M., Adolfsson-Erici M., McLachlan M.S., Sandborgh-Englund G. (2006). Triclosan in plasma and milk from Swedish nursing mothers and their exposure via personal care products. Sci. Total Environ..

[12] Wolff M.S., Teitelbaum S.L., Windham G., Pinney S.M., Britton J.A., Chelimo C., Godbold J., Biro F., Kushi L.H., Pfeiffer C.M. (2007). Pilot study of urinary biomarkers of phytoestrogens, phthalates, and phenols in girls. Environ. Health Perspect..

[13] Calafat A.M., Kuklenyik Z., Reidy J.A., Caudill S.P., Ekong J., Needham L.L. (2005). Urinary concentrations of bisphenol A and 4-nonylphenol in a human reference population. Environ. Health Perspect..

[14] Calafat A.M., Ye X., Wong L.Y., Reidy J.A., Needham L.L. (2008). Exposure of the US population to bisphenol a and 4-tertiary-octylphenol: 2003–2004. Environ. Health Perspect..

[15] Yang M., Kim S.Y., Lee S.M., Chang S.S., Kawamoto T., Jang J.Y., Ahn Y.O. (2003). Biological monitoring of bisphenol a in a Korean population. Arch. Environ. Contam. Toxicol..

[16] Sun Y., Irie M., Kishikawa N., Wada M., Kuroda N., Nakashima K. (2004). Determination of bisphenol A in human breast milk by HPLC with column-switching and fluorescence detection. Biomed. Chromatogr..

[17] Kuroda N., Kinoshita Y., Sun Y., Wada M., Kishikawa N., Nakashima K., Makino T., Nakazawa H. (2003). Measurement of bisphenol A levels in human blood serum and ascitic fluid by HPLC using a fluorescent labeling reagent. J. Pharm. Biomed. Anal..

[18] Calafat A.M., Wong L.Y., Ye X., Reidy J.A., Needham L.L. (2008). Concentrations of the sunscreen agent benzophenone-3 in residents of the United States: National Health and Nutrition Examination Survey 2003–2004. Environ. Health Perspect..

[19] Ye X., Kuklenyik Z., Needham L.L., Calafat A.M. (2006). Measuring environmental phenols and chlorinated organic chemicals in breast milk using automated on-line column-switching-high performance liquid chromatography-isotope dilution tandem mass spectrometry. J. Chromatogr. B.

[20] Janjua N.R., Kongshoj B., Andersson A.M., Wulf H.C. (2008). Sunscreens in human plasma and urine after repeated whole-body topical application. J. Eur. Acad. Dermatol. Venereol..

[21] Kolpin D.W., Furlong E.T., Meyer M.T., Thurman E.M., Zaugg S.D., Barber L.B., Buxton H.T. (2002). Pharmaceuticals, hormones, and other organic wastewater contaminants in U.S. streams, 1999–2000: A national reconnaissance. Environ. Sci. Technol..

[22] Singer H., Müller S., Tixier C., Pillonel L. (2002). Triclosan: Occurrence and fate of a widely used biocide in the aquatic environment: Field measurements in wastewater treatment plants, surface waters, and lake sediments. Environ. Sci. Technol..

[23] Yang G.C., Tsai H.J., Chang F.K. (2015). Occurrence of triclosan in the tropical rivers receiving the effluents from the hospital wastewater treatment plant. Environ. Monit. Assess..

[24] Balmer M.E., Buser H.R., Müller M.D., Poiger T. (2005). Occurrence of some organic UV filters in wastewater, in surface waters, and in fish from Swiss lakes. Environ. Sci. Technol..

[25] Cuderman P., Heath E. (2007). Determination of UV filters and antimicrobial agents in environmental water samples. Anal. Bioanal. Chem..

[26] Kameda Y., Kimura K., Miyazaki M. (2011). Occurrence and profiles of organic sun-blocking agents in surface waters and sediments in Japanese rivers and lakes. Environ. Pollut..

[27] Rudel R.A., Melly S.J., Geno P.W., Sun G., Brody J.G. (1998). Identification of alkylphenols and other estrogenic phenolic compounds in wastewater, septage, and groundwater on Cape Cod, Massachusetts. Environ. Sci. Technol..

[28] Ding W.H., Wu C.Y. (2000). Determination of estrogenic nonylphenol and bisphenol A in river water by solid-phase extraction and gas chromatography–mass spectrometry. J. Chin. Chem. Soc-Taip..

[29] Bolz U., Hagenmaier H., Komer W. (2001). Phenolic xenoestrogens in surface water, sediments and sewage sludge from Baden-Wurttemberg, South-west Germany. Environ. Pollut..

[30] Matthews J.B., Twomey K., Zacharewski T.R. (2001). In vitro and in vivo interactions of bisphenol A and its metabolite, bisphenol a glucuronide, with estrogen receptors alpha and beta. Chem. Res. Toxicol..

[31] Jung E.M., An B.S., Choi K.C., Jeung E.B. (2012). Potential estrogenic activity of triclosan in the uterus of immature rats and rat pituitary GH3 cells. Toxicol. Lett..

[32] Schlumpf M., Cotton B., Conscience M., Haller V., Steinmann B., Lichtensteiger W. (2001). In vitro and in vivo estrogenicity of UV screens. Environ. Health Perspect..

[33] Kunz P.Y., Fent K. (2006). Multiple hormonal activities of UV filters and comparison of in vivo and in vitro estrogenic activity of ethyl-4-aminobenzoate in fish. Aquat. Toxicol..

[34] Ma R., Cotton B., Lichtensteiger W., Schlumpf M. (2003). UV filters with antagonistic action at androgen receptors in the MDA-kb2 cell transcriptional-activation assay. Toxicol. Sci..

[35] Howdeshell K.L., Hotchkiss A.K., Thayer K.A., Vandenbergh J.G., vom Saal F.S. (1999). Environmental toxins: Exposure to bisphenol a advances puberty. Nature.

[36] Caserta D., Segni N.D., Mallozzi M., Giovanale V., Mantovani A., Marci R., Moscarini M. (2014). Bisphenol A and the female reproductive tract: An overview of recent laboratory evidence and epidemiological studies. Reprod. Biol. Endocrinol..

[37] Mallozzi M., Leone C., Manurita F., Bellati F., Caserta D. (2017). Endocrine Disrupting Chemicals and Endometrial Cancer: An Overview of Recent Laboratory Evidence and Epidemiological Studies. Int. J. Environ. Res. Public Health.

[38] Mallozzi M., Bordi G., Garo C., Caserta D. (2016). The effect of maternal exposure to endocrine disrupting chemicals on fetal and neonatal development: A review on the major concerns. Birth Defects Res. C Embryo Today.

[39] Timms B.G., Howdeshell K.L., Barton L., Bradley S., Richter C.A., vom Saal F.S. (2005). Estrogenic chemicals in plastic and oral contraceptives disrupt development of the fetal mouse prostate and urethra. Proc. Natl. Acad. Sci. USA.

[40] Markey C.M., Luque E.H., de Toro M.M., Sonnenschein C., Soto A.M. (2001). In utero exposure to bisphenol A alters the development and tissue organization of the mouse mammary gland. Biol. Reprod..

[41] Vom Saal F.S., Cooke P.S., Buchanan D.L., Palanza P., Thayer K.A., Nagel S.C., Parmigiani S., Welshons W.V. (1998). A physiologically based approach to the study of bisphenol A and other estrogenic chemicals on the size of reproductive organs, daily sperm production, and behavior. Toxicol. Ind. Health.

[42] Richter C.A., Birnbaum L.S., Farabollini F., Newbold R.R., Rubin B.S., Talsness C.E., Vandenbergh J.G., Walser-Kuntz D.R., vom Saal F.S. (2007). In vivo effects of bisphenol A in laboratory rodent studies. Reprod. Toxicol..

[43] Veldhoen N., Skirrow R.C., Osachoff H., Wigmore H., Clapson D.J., Gunderson M.P., Aggelen G.V., Helbing C.C. (2006). The bactericidal agent triclosan modulates thyroid hormone-associated gene expression and disrupts postembryonic anuran development. Aquat. Toxicol..

[44] Kumar V., Chakraborty A., Kural M.R., Roy P. (2009). Alteration of testicular steroidogenesis and histopathology of reproductive system in male rats treated with triclosan. Reprod. Toxicol..

[45] Coronado M., De Haro H., Deng X., Rempel M.A., Lavado R., Schlenk D. (2008). Estrogenic activity and reproductive effects of the UV-filter oxybenzone (2-hydroxy-4-methoxyphenyl-methanone) in fish. Aquat. Toxicol..

[46] Blüthgen N., Zucchi S., Fent K. (2012). Effects of the UV filter benzophenone-3 (oxybenzone) at low concentrations in zebrafish (Danio rerio). Toxicol. Appl. Pharmacol..

[47] Philippat C., Mortamais M., Chevrier C., Petit C., Calafat A.M., Ye X., Silva M.J., Brambilla C., Pin I., Charles M.A. (2012). Exposure to phthalates and phenols during pregnancy and offspring size at birth. Environ. Health Perspect..

[48] Wolff M.S., Engel S.M., Berkowitz G.S., Ye X., Silva M.J., Zhu C., Wetmur J., Calafat A.M. (2008). Prenatal phenol and phthalate exposures and birth outcomes. Environ. Health Perspect..

[49] Koeppe E.S., Ferguson K.K., Colacino J.A., Meeker J.D. (2013). Relationship between urinary triclosan and paraben concentrations and serum thyroid measures in NHANES 2007–2008. Sci. Total Environ..

[50] Tsutsumi O. (2005). Assessment of human contamination of estrogenic endocrine-disrupting chemicals and their risk for human reproduction. J. Steroid Biochem. Mol. Biol..

[51] Janjua N.R., Mogensen B., Andersson A.M., Petersen J.H., Henriksen M., Skakkebæk N.E., Wulf H.C. (2004). Systemic absorption of the sunscreens benzophenone-3, octyl-methoxycinnamate, and 3-(4-methyl-benzylidene) camphor after whole-body topical application and reproductive hormone levels in humans. J. Investig. Dermatol..

[52] Ye X., Kuklenyik Z., Needham L.L., Calafat A.M. (2005). Automated on-line column-switching HPLC-MS/MS method with peak focusing for the determination of nine environmental phenols in urine. Anal. Chem..

[53] Li X., Ying G.G., Zhao J.L., Chen Z.F., Lai H.J., Su H.C. (2013). 4-Nonylphenol, bisphenol-A and triclosan levels in human urine of children and students in China, and the effects of drinking these bottled materials on the levels. Environ. Int..

[54] Frederiksen H., Aksglaede L., Sorensen K., Nielsen O., Main K.M., Skakkebaek N.E., Andersson A.M. (2013). Bisphenol A and other phenols in urine from Danish children and adolescents analyzed by isotope diluted TurboFlow-LC–MS/MS. Int. J. Hyg. Environ. Health.

[55] Kim K., Park H., Yang W., Lee J.H. (2011). Urinary concentrations of bisphenol A and triclosan and associations with demographic factors in the Korean population. Environ. Res..

[56] Becker K., Güen T., Seiwert M., Conrad A., Pick-Fuß H., Müller J., Wittassek M., Schulz C., Kolossa-Gehring M. (2009). GerES IV: Phthalate metabolites and bisphenol A in urine of German children. Int. J. Hyg. Environ. Health.

[57] He Y., Miao M., Herrinton L.J., Wu C., Yuan W., Zhou Z., Li D.K. (2009). Bisphenol A levels in blood and urine in a Chinese population and the personal factors affecting the levels. Environ. Res..

[58] Zhao J.L., Ying G.G., Liu Y.S., Chen F., Yang J.F., Wang L. (2010). Occurrence and risks of triclosan and triclocarban in the Pearl River system, South China: From source to the receiving environment. J. Hazard. Mater..

[59] Geens T., Neels H., Covaci A. (2009). Sensitive and selective method for the determination of bisphenol-A and triclosan in serum and urine as pentafluorobenzoate-derivatives using GC–ECNI/MS. J. Chromatogr. B.

[60] Kim Y.H., Kim C.S., Park S., Han S.Y., Pyo M.Y., Yang M. (2003). Gender differences in the levels of bisphenol A metabolites in urine. Biochem. Biophys. Res. Commun..

